# Comparison of 2-hour drip infusions of meropenem versus piperacillin/tazobactam for febrile neutropenia in pediatric patients

**DOI:** 10.1128/aac.01608-24

**Published:** 2025-07-08

**Authors:** Ryoji Kobayashi, Hirozumi Sano, Ryusuke Ishigaki, Satoru Matsushima, Daiki Hori, Masato Yanagi, Daisuke Suzuki

**Affiliations:** 1 Department of Hematology/Oncology for Children and Adolescents, Sapporo Hokuyu Hospital 73962, Sapporo, Japan; The Peter Doherty Institute for Infection and Immunity, Melbourne, Victoria, Australia

**Keywords:** meropenem, piperacillin/tazobactam, febrile neutropenia, two-hour infusion

## Abstract

Although the treatment of pediatric hematological neoplastic diseases has improved greatly, the incidence of febrile neutropenia (FN) is more frequent and sometimes proves fatal as chemotherapy is intensified. A prospective, randomized study was performed to compare the usefulness of 2-hour drip infusions of meropenem (MEPM) and piperacillin/tazobactam (PIPC/TAZ) for pediatric patients with FN. Ninety-three patients with 405 febrile episodes were randomly assigned to receive MEPM or PIPC/TAZ. MEPM 120 mg/kg/day was administered as a 2-hour drip infusion three times a day, whereas PIPC/TAZ 360 mg/kg/day was administered as a 2-hour drip infusion four times a day. In addition, in cases that failed first-line therapy, the antibiotic administration was alternated and randomized with or without intravenous immunoglobulin (IVIG) as the second-line treatment. As the first-line treatment, MEPM was effective in 74.8% of the 206 episodes, and PIPC/TAZ was effective in 74.9% of the 199 episodes. The total success rate in second-line treatment was 65.6%. MEPM was effective in 70.8% of the 48 episodes, and PIPC/TAZ was effective in 60.4% of the 48 episodes. The combined first- and second-line treatment efficacy rates were 89.4% for the first-line treatment with MEPM and the second-line treatment with PIPC/TAZ group and 91.3% in the first-line treatment with PIPC/TAZ and second-line treatment with MEPM groups, with an overall efficacy rate of 90.4%, an excellent result. However, the efficacy of IVIG could not be proven in this study. Adolescents and young adults in the present study also presented with lower rates of antibiotic efficacy.

## INTRODUCTION

In the treatment of hematological neoplastic diseases, overcoming infections is essential to improve therapeutic outcomes (1, 2). There have been a few reports of guidelines for treating febrile neutropenia (FN) in children (3, 4). Recently, monotherapy with an antipseudomonal β-lactam, a fourth-generation cephalosporin, or a carbapenem was recommended for treating high-risk FN in children. Piperacillin/tazobactam (PIPC/TAZ) was also recommended in a guideline for FN in adults (5). In children, several reports of the efficacies of PIPC/TAZ and MEPM have been published (6), and we previously reported several trials comparing the efficacy of the two antibiotics for treating pediatric FN (7–12). In addition, we have previously reported that intravenous immunoglobulin (IVIG) is effective in patients with low IgG levels (13). The most recent report was a comparison using meropenem (MEPM) 120 mg/kg/day as a 1-hour drip infusion three times a day and PIPC/TAZ 360 mg/kg/day as a 1-hour drip infusion four times a day, and the efficacies of MEPM and PIPC/TAZ were 69.5% and 77.2%, respectively (12). Therefore, according to pharmacokinetic (PK)/pharmacodynamic (PD) principles, the present study was planned to compare the efficacy of MEPM 120 mg/kg/day as a 2-hour drip infusion three times a day with that of PIPC/TAZ 360 mg/kg/day as a 2-hour drip infusion four times a day. To examine the efficacy of IVIG, IgG levels were examined and studied at the time of first-line enrollment. In addition, as the second-line treatment, efficacy with or without IVIG was examined, as well as changing the antibiotic. Furthermore, factors involved in the efficacy of the antibiotics were analyzed.

## MATERIALS AND METHODS

From April 2020 to March 2024, febrile and neutropenic children, adolescents, and young adults who had been treated with chemotherapy, immunosuppressive therapy, or had received stem cell transplantation in the pediatric unit at Sapporo Hokuyu Hospital were enrolled in this study. Patients were eligible if they satisfied the following criteria: (i) fever defined as a temperature ≥37.5°C for at least 1 hour or a single temperature ≥38°C; (ii) an absolute neutrophil count (ANC) of less than 0.5 × 10^9^ /L or an expected ANC decrease to less than 0.5 × 10^9^ /L in several days; and (iii) no antibiotics within 72 hours prior to the initiation of treatment, except for trimethoprim-sulfamethoxazole prophylaxis for *Pneumocystis jirovecii* pneumonia. Prophylactic oral voriconazole at 10 mg/kg per day (maximum 400 mg/day) was used for patients with acute myelogenous leukemia (AML), whereas oral fluconazole 10 mg/kg per day (maximum 400 mg/day) was used for all other patients. For stem cell transplantation patients, intravenous micafungin at 1 mg/kg per day (maximum 50 mg/day) was used from the beginning of the conditioning regimen to neutrophil recovery, followed by administration of oral fluconazole at 10 mg/kg per day (maximum 400 mg/day) from the time of neutrophil recovery to the date of discharge. Laboratory examinations consisting of complete blood counts, peripheral blood smears, quantitative C-reactive protein (CRP), liver and renal functions, urinalyses, and blood cultures from specimens obtained via a peripheral venous puncture or central venous access device, if in place, were performed. Patients were randomly allocated to the MEPM or PIPC/TAZ group. MEPM 120 mg/kg/day (maximum dose: 3 g/day) was administered as a 2-hour drip infusion three times a day, whereas PIPC/TAZ 360 mg/kg/day (maximum dose: 18 g/day) was administered as a 2-hour drip infusion four times a day. Randomization was performed using the envelope method. Patients were randomized when ANC was less than 0.5 × 10^9^ /L. If a febrile episode occurred, antibiotic therapy was started immediately, even at night or on a holiday. After using antibiotics, patients were randomized when ANC was less than 0.5 × 10^9^ /L as a new event in the same way. In all episodes, a follow-up was available. Severe adverse effects that should result in stopping the administration of antibiotics were excluded from the analysis of efficacy. Clinical efficacy was evaluated at 120 hours, with treatment outcome criteria defined as follows. Success was defined as the disappearance of fever, clinical improvement, eradication of the infecting organism, and maintenance of a response for at least 7 days after discontinuation of treatment. Failure was defined as persistence of fever or the infecting organism, any required modification of antibiotic therapy, new infections, or infection-related death. New infectious episodes (breakthrough infection) were defined as cases in which fever and infectious signs resolved transiently within 120 hours following initiation of treatment, with the subsequent recurrence of fever despite continuation of the same antibiotic therapy.

**Fig 1:**
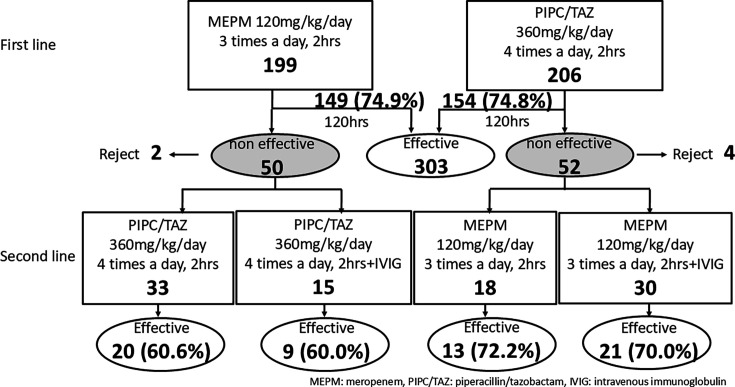
Study design, episode numbers, and results.

Second-line therapy was administered to patients who failed first-line treatment therapy. In second-line therapy, patients were randomized to a group of MEPM or PIPC/TAZ with or without IVIG at 100 mg/kg/day (maximum dose: 5 g/day) for 3 days (venoglobulin IH 5% I.V, Japan Blood Products Organization, Tokyo, Japan). MEPM 120 mg/kg/day (maximum dose: 3 g/day) was administered as a 2-hour drip infusion three times a day for patients who received PIPC/TAZ as the first-line treatment, whereas PIPC/TAZ 360 mg/kg/day (maximum dose: 18 g/day) was administered as a 2-hour drip infusion four times a day for patients who received MEPM as the first-line treatment (Fig. 1). A complete history; physical examination; laboratory tests; measurements of β-D glucan, presepsin, and procalcitonin levels; and blood cultures were performed as clinically indicated. The effect of the second-line therapy was evaluated 72 hours after the start of therapy. When second-line therapy was ascertained to have failed, routine chest and abdominal CT was also performed. Treatment was continued until completion of an appropriate course of therapy for a defined clinical or microbiological infection. This research was approved by the Institutional Review Board of our hospital (No. 2000312.02). Written, informed consent was obtained from all patients or their parents.

### Statistical analysis

Differences between groups were analyzed using Fisher’s exact test and the Mann-Whitney U test. Statistical analyses were performed using EZR (14) (free software).

## RESULTS

### First-line treatment

Ninety-three patients with 405 febrile neutropenic episodes were enrolled in this study. The patients had 1 to 18 febrile episodes. The median age of the patients was 10.0 years (range: 0–25 years). There were 51 male and 42 female patients. Most episodes occurred in patients with leukemia (61.1%), including acute lymphoblastic leukemia (ALL) (*n* = 39), acute myelomonocytic leukemia (AML) (*n* = 14), chronic myelomonocytic leukemia (CML) (*n* = 1), myelodysplastic syndrome (MDS) (*n* = 3), and juvenile myelomonocytic leukemia (*n* = 1); 10.5% of the patients had aplastic anemia (*n* = 10), and 28.4% of the total patients had solid tumors, including hepatoblastoma (*n* = 1), neuroblastoma (*n* = 1), non-Hodgkin’s lymphoma (*n* = 1). (*n* = 15), Hodgkin’s lymphoma (*n* = 2), Langerhans cell histiocytosis (*n* = 1), Ewing’s sarcoma (*n* = 1), ovary tumor (*n* = 1), colon cancer (*n* = 1), spindle cell sarcoma (*n* = 1), and brain tumor (*n* = 3). In 33 episodes, the patients had received stem cell transplantation. Three patients had Down’s syndrome (eight episodes). Two patients developed secondary cancers during the study, one from ALL to AML and the other from AML to MDS. Granulocyte colony-stimulating factor (G-CSF) was used in 12 episodes (PIPC/TAZ group 4; MEPM group 8), and all were used for stem cell transplantation and for aplastic anemia.

Results were analyzed on an episode-by-episode basis. Sex, age, original disease, and the condition of the patients were not different between the MEPM and PIPC/TAZ groups (Table 1). In 25 of 405 episodes, blood cultures were positive, and organisms isolated from blood cultures are shown in Table 2. The total success rate was 74.8%. MEPM was effective in 74.9% of the 199 episodes, and PIPC/TAZ was effective in 74.8% of the 206 episodes; the difference was not significant (Fig. 1). The success rate of patients with ANC < 0.5 × 10^9^ /L at the end of antibiotic treatment was 69.7% with MEPM and 67.7% with PIPC/TAZ, and this difference was not significant (*P* = 0.793). The success rate was 52.0% in all patients with positive blood cultures, whereas it was 76.3% in patients with negative blood cultures, and this difference was significant (*P* = 0.0147). Efficacy was not associated with species of detected bacteria. In patients with positive blood cultures, success rates were 63.6% in the MEPM group and 42.8% in the PIPC/TAZ group. In 12 episodes using G-CSF, only one episode was effective. Although the infection-related death rate was 0% in both groups, two deaths were observed during the second-line therapy. The efficacy rates by number of febrile neutropenic events in the study were 75.0%, 76.2%, 81.3%, and 72.7% for the first, second, third, and fourth or more events, respectively (Table 3). Adverse effects were observed in 113 episodes. Liver dysfunction was observed in 55 episodes; 29 episodes were of grade 3 (MEPM: 13 episodes, PIPC/TAZ: 8 episodes) using the Common Terminology Criteria for Adverse Events (CTCAE) version 5, and one episode was of grade 4 (PIPC/TAZ). The rate of adverse events of liver function was 15.5% in the MEPM group and 9.7% in the PIPC/TAZ group; this difference was not significant (Table 4). Diarrhea was observed in 66 episodes; 19 episodes were of grade 3 (MEPM 4 episodes, PIPC/TAZ: 15 episodes) using CTCAE version 5. The rate of incidence of adverse events of diarrhea was 9.5% in the MEPM group and 22.8% in the PIPC/TAZ group; this difference was statistically significant (*P* < 0.001). In four episodes, both adverse effects were observed.

**TABLE 1:** Demographic and clinical characteristics of patients with febrile neutropenia in the PIPC/TAZ and MEPM groups^*a*^

	PIPC/TAZ (*n* = 206)	MEPM (*n* = 199)	P
Sex (M/F)	111/95	97/102	0.321
Age (y, median, range)	10 (0–25)	10 (0–25)	0.828
Body weight (kg)	33.05 (3.09–85.2)	34.1 (2.96–87.7)	0.879
Disease			0.615
ALL	95	86	
AML	38	47	
AHL	26	18	
Solid tumor	17	17	
Aplastic anemia	12	16	
Other	18	15	
Stem cell transplantation	9	9	1.000
CV line	206	198	0.491
WBC at entry (×10^9^ /L, median, range)	0.665 (0.01–139.29)	0.68 (0.01–177.97)	0.764
CRP at entry (mg/dL, median, range)	0.375 (0.01–16.4)	0.27 (0.01–32.33)	0.173
IgG at entry (mg/dL, median, range)	715 (150–1,527)	806.5 (96-1,707)	0.195

^*a*^ PIPC/TAZ: piperacillin/tazobactam, MEPM: meropenem, M: male, F: female, ALL: acute lymphoblastic leukemia, AML: acute myelogenous leukemia, NHL: non-Hodgkin’s lymphoma, CV line: central venous line, WBC: white blood cell, CRP: C-reactive protein.

**TABLE 2:** Organisms isolated from blood cultures of febrile neutropenic patients^*a*^

	First-line		Second-line	
	PIPC/TAZ	MEPM	PIP/TAZ	MEPM
Microbiologically documented	14	11	3	4
*Staphylococcus aureus*	2	1	0	0
*Staphylococcus epidermidis*	4	0	3	0
*Streptococcus*	3	4	0	0
*Rothia*	2	0	0	0
*Micrococcus luteus*	0	0	0	1
*Klebsiella species*	1	4	0	0
*Pseudomonas aeruginosa*	1	0	0	2
*Stenotrophomonas maltophilia*	0	0	0	1
*Enterobacter cloacae*	1	0	0	0
*Bacillus species*	0	1	0	0
*Paenibacillus species*	0	1	0	0

^*a*^ PIPC/TAZ: piperacillin/tazobactam, MEPM: meropenem.

**TABLE 3:** Effectiveness of each treatment groups^*a*^

First line	N	All (*n* = 405)	PIPC/TAZ (*n* = 206)	MEPM (*n* = 199)
All episodes	405	303/405 (74.8%)	154/206 (74.8%)	149/199 (74.9%)
First episode of FN	90	71/90 (78.9%)	33/44 (75.0%)	38/46 (82.6%)
Second episode of FN	78	61/78 (78.2%)	32/42 (76.2%)	29/36 (80.6%)
Third episode of FN	62	51/62 (82.2%)	26/32 (81.3%)	25/30 (83.3%)
Fourth or over episode of FN	175	123/175 (70.3%)	64/88 (72.7%)	59/87 (67.8%)
Bacteremia	25	13/25 (52.0%)	6/14 (42.9%)	7/11 (63.6%)
No bacteremia	380	290/380 (76.3%)	148/192 (77.1%)	142/188 (75.5%)

Second line	N	All (*n* = 96)	MEPM as second line (*n* = 48)	PIPC/TAZ as second line (*n* = 48)
All episodes	96	63/96 (65.6%)	34/48 (70.8%)	29/48 (60.4%)
With IVIG	45	30/45 (66.7%)	21/30 (70.0%)	9/15 (60.0%)
Without IVIG	51	33/51 (64.7%)	13/18 (72.2%)	20/33 (60.6%)
Bacteremia	7	1/7 (14.3%)	1/4 (25.0%)	0/3 (0%)
No bacteremia	89	62/89 (69.7%)	33/44 (75.0%)	29/45 (64.4%)

First and second line	N	All (*n* = 399)	PIPC/TAZ as first line (*n* = 202)	MEPM as first line (*n* = 197)
First episode of FN	89	82/88 (93.2%)	40/42 (95.2%)	42/46 (91.3%)
Second episode of FN	78	71/78 (91.0%)	38/42 (90.5%)	33/36 (91.7%)
Third episode of FN	61	61/64 (95.3%)	31/31 (100%)	30/33 (90.9%)
Fourth or over episode of FN	172	155/172 (90.1%)	79/87 (90.8%)	76/85 (89.4%)
Bacteremia in first or second line	28	20/28 (71.4%)	11/15 (73.3%)	9/13 (69.2%)
No bacteremia	371	346/371 (93.3%)	177/187 (94.7%)	169/184 (91.8%)

^*a*^ PIPC/TAZ: piperacillin/tazobactam, MEPM: meropenem, FN: febrile neutropenia, IVIG: intravenous immunoglobulin.

**TABLE 4:** Adverse effects of the first-line treatment^*a*^

	PIPC/TAZ (*n* = 206)	MEPM (*n* = 199)	P
Liver dysfunction	20 (9.7%)	31 (15.6%)	0.099
Grade 1	9	12	
Grade 2	2	6	
Grade 3	8	13	
Grade 4	1	0	
Diarrhea	47 (22.8%)	19 (9.5%)	<0.001
Grade 1	16	6	
Grade 2	16	9	
Grade 3	15	4	
Grade 4	0	0	

^*a*^ PIPC/TAZ: piperacillin/tazobactam, MEPM: meropenem.

Compared with our previous study (12) involving administration of MEPM 120 mg/kg/day three times a day as a 1-hour drip infusion versus PIPC/TAZ 360 mg/kg/day four times a day as a 1-hour drip infusion, the success rates of the MEPM and PIPC/TAZ groups were not different (MEPM: previous study 69.5% versus present study 74.9%, *P* = 0.217, PIPC/TAZ: previous study 77.2% versus present study 74.8%, *P* = 0.725).

### Second-line treatment

There were 102 failed first-line treatment episodes, and 54 patients with 96 febrile neutropenic episodes were enrolled in the second-line treatment study. Six episodes were excluded due to the susceptibility of the detected bacteria or because they were thought to be tumor fever. The patients had 1 to 7 febrile episodes. The median age of the patients was 12.0 years (range: 0–23 years). There were 26 male and 28 female patients. Most episodes (63.0%) occurred in patients with leukemia, including ALL (*n* = 22), AML (*n* = 10), chronic myelomonocytic leukemia (*n* = 1), and juvenile myelomonocytic leukemia (*n* = 1); 11.1% of the total patients had aplastic anemia (*n* = 6), and 25.9% of the total patients had solid tumors, including patients with neuroblastoma (*n* = 1), non-Hodgkin’s lymphoma (*n* = 8), Ewing’s sarcoma (*n* = 1), Langerhans cell histiocytosis (*n* = 1), spindle cell sarcoma (*n* = 1), colon cancer (*n* = 1), and brain tumor (*n* = 1). In 10 episodes, the patients had received stem cell transplantation. One patient had Down’s syndrome (one episode). Intravenous antifungal drugs were administered as second-line treatment to 11 patients, including patients for whom it was used as a prophylaxis in stem cell transplantation, and granulocyte colony-stimulating factor (G-CSF) was administered to 10 patients.

Sex, age, original disease, and the condition of the patients were not different between the MEPM and PIPC/TAZ groups (Table 5). However, CRP and presepsin levels were higher in the MEPM group. In 7 of 96 episodes, blood cultures were positive, and organisms isolated from blood culture were coagulase-negative *Staphylococci* in three, *Micrococcus luteus* in one, *Pseudomonas aeruginosa* in two, and *Stenotrophomonas maltophilia* in one (Table 2). Only one episode of positive blood culture was detected in both first- and second-line treatment patients, and the organism detected was *Pseudomonas aeruginosa*. The total success rate in second-line treatment was 65.6%. MEPM was effective in 70.8% of the 48 episodes, and PIPC/TAZ was effective in 60.4% of the 48 episodes; the difference was not significant. The success rate of patients with ANC < 0.5 × 10^9^ /L at the end of the antibiotic treatment was 48.0% with MEPM and 45.8% with PIPC/TAZ. In seven episodes with positive blood cultures at the start of the second-line treatment, only one episode with *Micrococcus luteus* was effective. Infection-related deaths were observed in two episodes, and these episodes were in the MEPM group.

**TABLE 5:** Demographic and clinical characteristics of patients with febrile neutropenia in the PIPC/TAZ and MEPM groups and with or without IVIG in the second-line treatment group^*a*^

Second-line	PIPC/TAZ (*n* = 48)	MEPM (*n* = 48)	*P*	IVIG (*n* = 33)	No-IVIG (*n* = 63)	*P*
Sex (M/F)	18/30	27/21	0.101	17/16	28/35	0.527
Age (y, median, range)	10 (0–23)	13 (1–23)	0.124	13 (1–23)	10 (0–23)	0.646
Disease			0.789			0.497
ALL	18	18		11	25	
AML	15	13		13	15	
Other leukemia	3	2		1	4	
NHL	3	7		3	7	
Solid tumor	4	5		4	5	
Aplastic anemia	5	3		1	7	
Condition of patients			0.816			0.418
Induction chemotherapy	10	10		5	15	
Complete remission	22	25		20	27	
Non-CR	7	5		4	9	
Stem cell transplantation	7	6	1.000	4	9	1.000
PIPC/TAZ / MEPM	-	-		15/18	33/30	0.668
IVIG +/–	15/33	18/30	0.668	-	-	
WBC at second-line entry (×10^9^ /L, median, range)	0.51 (0.01–48.4)	0.28 (0.01–4.5)	0.222	0.12 (0.01–6.35)	0.51 (0.01–48.4)	0.122
CRP at second-line entry (mg/dL, median, range)	1.70 (0.01–29.42)	4.36 (0.14–21.68)	0.012	4.49 (0.02–29.42)	2.55 (0.01–17.65)	0.129
IgG at first-line entry (mg/dL, median, range)	910 (96–1,338)	788 (262–1,462)	0.604	881.5 (262-1,283)	809 (96–1,462)	0.616
Procalcitonin at second-line entry (ng/mL)	0.13 (0.02–8.0)	0.24 (0.02–28.37)	0.111	0.25 (0.05–28.37)	0.165 (0.02–5.85)	0.208
Presepsin at second-line entry (pg/mL)	212 (66.8–1,461)	305 (104–1,334)	0.033	288 (99.8–1,228)	251.5 (66.8–1,461)	0.199
β-D glucan at second-line entry (pg/mL)	4.60 (0.4–566)	5.35 (2.3–14.5)	0.419	5.8 (0.4–566)	5 (2.3–23.8)	0.499
Bacteremia at first-line	2	5	0.435	3	4	0.689
Bacteremia at second-line	3	4	1.000	2	5	1.000

^*a*^ PIPC/TAZ: piperacillin/tazobactam, MEPM: meropenem, IVIG: intravenous immunoglobulin, M: male, F: female, ALL: acute lymphoblastic leukemia, AML: acute myelogenous leukemia, NHL: non-Hodgkin’s lymphoma, CV line: central venous line, WBC: white blood cell, CRP: C-reactive protein.

In addition, 33 episodes were allocated to the IVIG group and 63 to the non-IVIG group. Sex, age, original disease, and the condition of the patients were not different between the two groups (Table 5). The IVIG group showed effectiveness in 66.7% of the 33 episodes, and the non-IVIG group showed effectiveness in 65.1% of the 63 episodes. The success rate of patients with absolute neutrophil counts <0.5 × 10^9^ /L at the end of antibiotic treatment was 36.4% in the IVIG group and 50.0% in the non-IVIG group. In episodes where less than 500 mg/dL of IgG was administered at the start of the first-line treatment, the success rates were 57.7% in the IVIG group and 76.9% in the non-IVIG group. None of these differences were significant. Specifically, the efficacy rates were 60.6% for the PIPC/TAZ group without IVIG, 60.0% for the PIPC/TAZ group with IVIG, 72.2% for the MEPM group without IVIG, and 70.0% for the MEPM group with IVIG.

### Overall result

**Fig 2:**
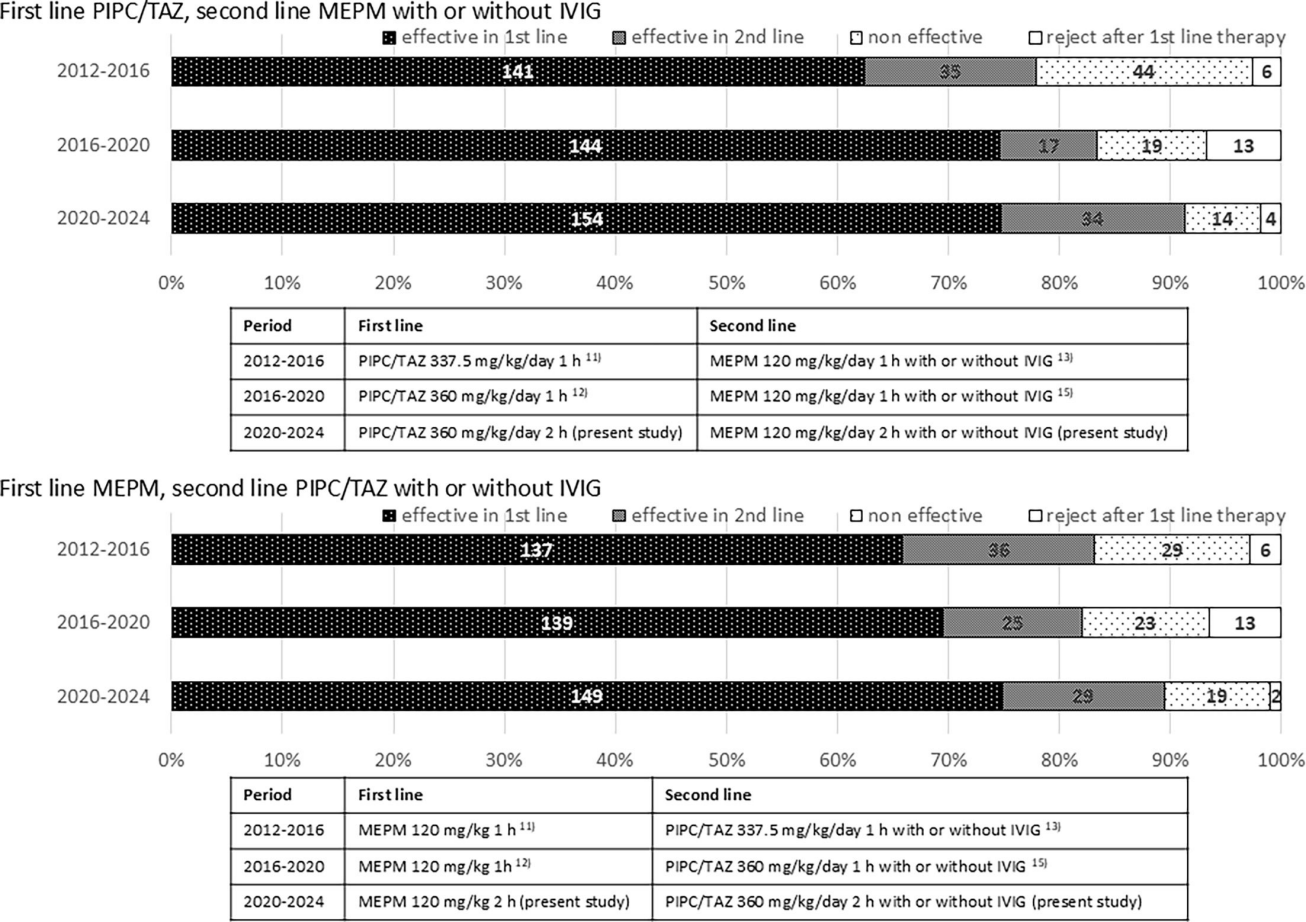
Comparison of results of the present and past studies.

The overall result with first-line and second-line treatments was 90.4% for all episodes. In episodes with PIPC/TAZ as first-line treatment and MEPM with or without IVIG as second-line treatment, the efficacy was seen in 91.3%, and in those with MEPM as first-line treatment and PIPC/TAZ with or without IVIG, the efficacy was seen in 89.4%, although the difference was not significant. Efficacy was significantly improved compared with that in our previous studies: PIPC/TAZ 360 mg/kg/day four times a day 1-hour drip vs MEPM 120 mg/kg/day three times a day 1-hour drip, overall success rate 82.7% (*P* = 0.0018) (12, 15); and PIPC/TAZ 337.5 mg/kg/day three times a day 1-hour drip vs MEPM 120 mg/kg/day three times a day 1-hour drip, overall success rate 80.4% (*P* < 0.001) (11, 13) (Fig. 2).

### Analysis of factors associated with success

Factors associated with treatment success were analyzed (Table 6). In the first-line treatment, non-effective episodes were associated with older age, heavier body weight, lower white blood cell (WBC) count, higher CRP level at entry, stem cell transplantation, and bacteremia. Although the same factors were associated in first- and second-line treatment, the WBC count was not associated with success. Receiver-operating characteristic (ROC) curve analysis showed age and weight cut-offs at 16 years and 52 kg, respectively. In episodes in patients under 16 years of age, the success rate in first- and second-line treatment was 94.0%, whereas in episodes in those 16 years and older, the success rate was 82.0%; this difference was significant (*P* < 0.001). Furthermore, in episodes in patients with weight under 52 kg, the success rate in first- and second-line treatment was 94.0%, whereas in patients with weight 52 kg and over, the success rate was 82.7%; this difference was significant (*P* = 0.002).

**TABLE 6:** Comparison of factors related to effectiveness in first-line and first- and second-line treatments^*a*^

	First-line treatment (*n* = 405)			First- and second-line treatment (*n* = 399)		
	Effective (*n* = 303)	Not effective (*n* = 102)	p	Effective (*n* = 366)	Not effective (*n* = 33)	*P*
Sex (M/F)	159/144	49/53	0.492	188/178	16/17	0.856
Age (y, median, range)	10 (0–25)	13 (0–23)	0.005	10 (0–25)	15 (3–23)	0.009
Body weight (kg)	28.6 (3.0–87.7)	37.9 (8.2–85.2)	0.006	32.15 (3.0–87.7)	44.85 (13.25–85.2)	0.032
Malignancy	283 (93.3%)	94 (92.2%)	0.656	343 (93.7%)	28 (84.8%)	0.070
Stem cell transplantation	8 (2.6%)	11 (10.8%)	0.002	12 (3.3%)	6 (18.2%)	0.002
WBC at entry (×10^9^ /L, median, range)	0.71 (0.01–177.97)	0.415 (0.01–139.29)	0.002	0.68 (0.01–177.97)	0.48 (0.01–100.78)	0.342
ANC at entry (×10^9^ /L, median, range)	0.102 (0–58,521)	0.052 (0–21,668)	0.059	0.093 (0–58,521)	0.063 (0–21,668)	0.797
CRP at entry (mg/dL, median, range)	0.27 (0.01–16.00)	0.62 (0.01–32.33)	<0.001	0.295 (0.01–16.00)	0.72 (0.02–32.33)	0.027
IgG at entry (mg/dL, median, range)	744 (119–1,707)	823.5 (96-1,462)	0.105	748 (119–1,707)	906 (96–1,462)	0.128
MEPM vs PIPC/TAZ used as first-line therapy	149/154	50/52	1.000	178/188	19/14	0.366
Bacteremia at first-line therapy	13 (4.3%)	12 (11.8%)	0.015	19 (5.2%)	5 (15.2%)	0.038
IVIG (second line)	-	-		30 (*n* = 45)	15 (*n* = 45)	

^*a*^ M: male, F: female, WBC: white blood cell, ANC: absolute neutrophil count, CRP: C-reactive protein, PIPC/TAZ: piperacillin/tazobactam, MEPM: meropenem, IVIG: intravenous immunoglobulin.

## DISCUSSION

According to pharmacokinetics/pharmacodynamic (PK/PD) principles, the efficacies of penicillin, cephalosporins, and carbapenems are associated with the time above the minimum inhibitory concentration (MIC) (16); the time above the MIC increases with the frequency of drug administration and duration of exposure. Several reports have examined the administration of PIPC/TAZ to children, as well as adults (17–20). The ultimate in this theory is 24 hour dosing, but there is a question as to whether the longer the dosing time, the lower the peak value and, therefore, the weaker the effect. In fact, there are reports that continuous 24 hour dosing did not differ in efficacy from intermittent dosing (21). It is not yet clear how long dosing times are useful in actual clinical practice. In the present study, the usefulness of PIPC/TAZ and MEPM with a dosing time of 2 hours was examined. The results were compared with those of our previous studies using 1-hour drip infusions. A further second line of treatment, also with a 2-hour drip infusion, was studied with and without IVIG and PIPC/TAZ and MEPM. We previously reported the efficacy of IVIG administration in cases with low IgG levels, and the success rate of MEPM or PIPC/TAZ with IVIG in patients with serum IgG under 1,000 mg/dL was higher than in those without IVIG (13). In the present study, IgG was measured at the first-line treatment, and the relationship with the efficacy of antibiotics of both first-line and second-line treatments was examined.

In the present study, PIPC/TAZ and MEPM were administered for 2 hours as the first-line treatment, with no significant difference between the two groups and no significant difference in efficacy rates compared with the previous study. There was also no relationship between IgG levels and the efficacy of antibiotic administration. However, the efficacy rate of the first-line treatment was extremely high, at around 75%. Therefore, it was difficult to evaluate IgG levels, and second-line treatment evaluation was also difficult since few patients progressed to the second-line treatment. There was no difference in efficacy between the PIPC/TAZ and MEPM groups in the second-line treatment and no superiority of IVIG administration. Unfortunately, due to the small number of cases, there was also no superiority of IVIG in patients with low IgG levels. However, the combined first- and second-line treatment total efficacy rate was greater than 90%, which was significantly better than those in previous studies. These results provide great encouragement for overcoming infections when administering chemotherapy. Unfortunately, however, two patients died of infection, and research into the treatment of infectious diseases must continue.

Factors associated with treatment success were also examined. Both in first-line and first- and second-line treatments, non-effective episodes were associated with higher age, heavier body weight, higher CRP level at entry, stem cell transplantation, and bacteremia. Higher CRP levels and bacteremia were expected. Moreover, fever during stem cell transplantation may sometimes be associated with noninfectious complications such as graft versus host disease and engraftment syndrome. In the past, we reported that the success rate of antibiotics was inferior in adolescents than in children (22). There were no differences in efficacy rates by age or weight at first-line treatment in the previous study (12). However, there were significant differences in overall efficacy rates for first-line and second-line combined treatment (data not shown). However, the present study demonstrated that, in both first-line and overall first- and second-line treatment results, older and heavier individuals had significantly lower efficacy rates. Older and heavier individuals are at the upper end of the antibiotic dosage range, resulting in lower doses per body weight. However, it is known that invasive fungal infection is more likely to occur at older ages, so there may be another factor. The cause of this is not clear, and further investigation is considered necessary. It has been reported that adolescent and young adult patients with ALL and AML have lower survival rates due to infection, even when treated with the same protocol as children (23, 24). Therefore, it is critical to understand why antibiotic efficacy is lower in adolescent and young adult patients.

There were several limitations in the present study: the analysis was performed on a fever episode basis rather than a patient basis. However, we chose to analyze each episode because the situation may differ depending on the treatment status of the same patient. Second, the results of the present study were compared with those of previous cases; this was not a randomized study. However, we have previously carried out similar studies in different phases, so comparisons may be possible. Third, although the sample size was large for a pediatric study, it was small compared with that for an adult study. Moreover, it was difficult to make sufficient comparisons due to the large number of second-line allocations. However, this was partly due to the improved results of the first-line treatment, which was unavoidable. Fourth, the number of cases in the IVIG and no IVIG groups differed in the second line due to an error in predicting the number of cases. Care must be taken to avoid this in future studies.

In conclusion, PIPC/TAZ 360 mg/kg/day and MEPM 120 mg/kg/day as 2-hour drip infusions were very effective for treating FN in children and adolescents. In addition, the combined success rate of the first- and second-lines was greater than 90%, an excellent result. However, this study did not demonstrate the efficacy of IVIG. Efficacy in older children was also low in the present study, and the reasons for this need to be clarified.
